# Exonic DNA Sequencing of *ERBB4* in Bipolar Disorder

**DOI:** 10.1371/journal.pone.0020242

**Published:** 2011-05-26

**Authors:** Fernando S. Goes, Michael Rongione, Yun-Ching Chen, Rachel Karchin, Eran Elhaik, the Bipolar Genome Study, James B. Potash

**Affiliations:** 1 Department of Psychiatry and Behavioral Sciences, Johns Hopkins School of Medicine, Baltimore, Maryland, United States of America; 2 Department of Biomedical Engineering and the Institute for Computational Medicine, Johns Hopkins University, Baltimore, Maryland, United States of America; 3 McKusick-Nathans Institute of Genetic Medicine, Johns Hopkins School of Medicine, Baltimore, Maryland, United States of America; University of Muenster, Germany

## Abstract

The Neuregulin-ErbB4 pathway plays a crucial role in brain development and constitutes one of the most biologically plausible signaling pathways implicated in schizophrenia and, to a lesser extent, in bipolar disorder (BP). However, recent genome-wide association analyses have not provided evidence for common variation in *NRG1* or *ERBB4* influencing schizophrenia or bipolar disorder susceptibility. In this study, we investigate the role of rare coding variants in *ERBB4* in BP cases with mood-incongruent psychotic features, a form of BP with arguably the greatest phenotypic overlap with schizophrenia. We performed Sanger sequencing of all 28 exons in *ERBB4*, as well as part of the promoter and part of the 3′UTR sequence, hypothesizing that rare deleterious variants would be found in 188 cases with mood-incongruent psychosis from the GAIN BP study. We found 42 variants, of which 16 were novel, although none were non-synonymous or clearly deleterious. One of the novel variants, present in 11.2% of cases, is located next to an alternative stop codon, which is associated with a shortened transcript of *ERBB4* that is not translated. We genotyped this variant in the GAIN BP case-control samples and found a marginally significant association with mood-incongruent psychotic BP compared with controls (additive model: OR = 1.64, *P*-value = 0.055; dominant model: OR = 1.73. *P*-valu*e* = 0.039). In conclusion, we found no rare variants of clear deleterious effect, but did uncover a modestly associated novel variant that could affect alternative splicing of *ERBB4*. However, the modest sample size in this study cannot definitively rule out a role for rare variants in bipolar disorder and studies with larger sample sizes are needed to confirm the observed association.

## Introduction

Mounting evidence suggests that the major psychoses share, to some degree, a common genetic susceptibility [1]–[3]. In particular, we and others have proposed that within the broad bipolar disorder spectrum, the subtype of bipolar disorder with mood-incongruent psychotic features is likely to be the subphenotype most closely aligned with schizophrenia [4], [5]. Among candidate genes implicated in the pathogenesis of both psychotic disorders, those in the Neuregulin1-ErbB4 signaling pathway have been frequently supported by association, copy number variation, and expression studies [6], although these findings have not always been consistent.

In the CNS, NRG1 functions primarily as a signaling molecule that binds to ERBB4, a tyrosine kinase receptor predominantly expressed in inhibitory neurons [7]. The *ERBB4* gene spans over 1.16 Mb and consists of 28 exons. Alternative splicing of exons 15/16 and exon 26 results in the formation of at least four protein isoforms that differ in their susceptibility to extra-cellular and intracellular cleavage [8]. Bound ERBB4 auto-phosphorylates several intracellular tyrosine residues, leading to the activation of key intracellular messengers such as phosphatidyl-inositol 3-kinase (PI3K) and AKT [9]. These proteins, among many functions, inhibit glycogen synthase kinase-3 (GSK-3), which is arguably the protein most strongly implicated in lithium's mechanism of action [10]. Post-mortem studies have also provided initial evidence for a functional interaction of ERBB4 with the NMDA receptor [11], also a promising therapeutic target for both mood disorders and schizophrenia.

Although *ERBB4* has been far less studied than *NRG1*, preliminary evidence suggests a possible association with schizophrenia. Among the initial candidate gene studies of *ERBB4*, Silberberg et al. found an association between schizophrenia and three highly linked markers surrounding exon 3 of the gene (best allelic *P*-value = 0.0049) [12]. However, the sample size was small (total *N* = 199) and these findings have not been replicated. Subsequent studies have focused on the interactions between *NRG1* and *ERBB4*; while significant interactions between various markers were reported by each study, there was little overlap among the actual interacting markers across studies [13]–[16]. Moreover, in genome-wide association studies (GWAS) of Caucasians with schizophrenia or bipolar disorder, neither *NRG1* nor *ERBB4* have featured among the top hits in the original studies or in subsequent meta-analyses [17], [18]. By contrast, the most highly associated SNP in the only GWAS of schizophrenia in African-Americans was found in *ERBB4* (rs1851196, *P*-value = 2.14×10^−6^), though the sample size was relatively modest by GWAS standards and the findings fell short of genome-wide significance.

The above studies of *ERBB4* have focused almost exclusively on common variants. However, increasing evidence indicates that rare variants might also play a role in the etiology of complex diseases such as bipolar disorder and schizophrenia [19]. Indeed, a large deletion (∼400 kb) of the 3′ region of *ERBB4* has been reported in a subject with schizophrenia [20], but, to our knowledge, no study has performed comprehensive sequencing of *ERBB4*.

While most of the evidence for association in the NRG1-ERBB4 pathway comes from studies of schizophrenia, we hypothesized that variation in the pathway might also be involved in susceptibility to bipolar disorder with mood-incongruent psychosis, where symptoms can often be indistinguishable from those of schizophrenia. In this study we have performed comprehensive sequencing of all 28 exons of *ERBB4* in cases with mood-incongruent psychosis from the GAIN BP sample [21]. While we did not find an excess of functional rare variants, we discovered a novel, potentially functional, common variant, which was additionally genotyped in a case-control association experiment. We demonstrate a modest excess of this variant that appears to be specific to the mood-incongruent form of BP.

## Results

**Figure 1:**
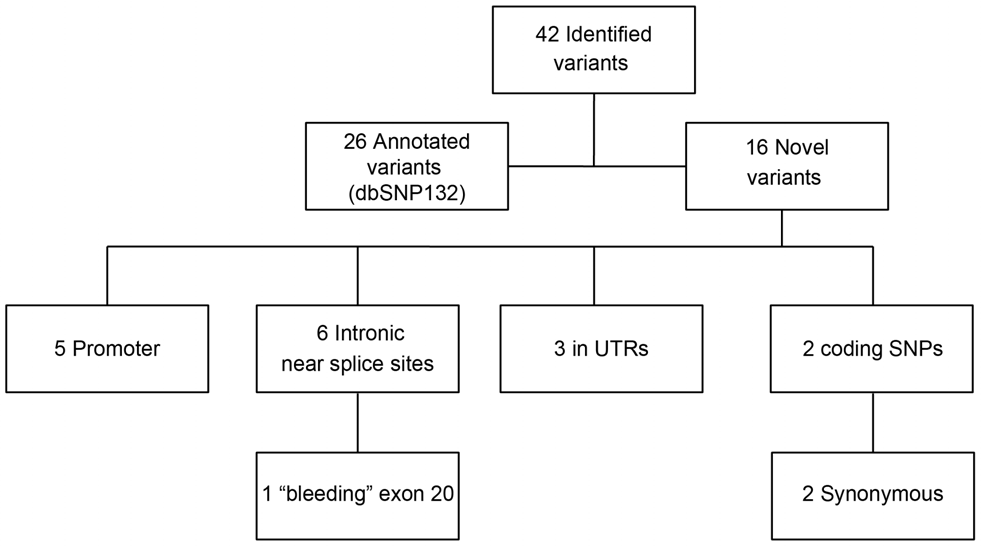
Variants identified by sequencing in *ERBB4*.

We analyzed 5.9 kb of DNA sequence representing all the 28 exons and surrounding sequences, as well as approximately 600 bp of the promoter sequence, all the 5′ UTR, and 400 bp of the 3′ UTR sequence in 188 BP subjects with mood-incongruent psychotic features. We found 42 variants; of these, 26 were single nucleotide variants (SNVs) present in dbSNP 132, including 17 common polymorphisms present in the HapMap CEU sample (Fig. 1). We discovered 16 novel variants across *ERBB4* (Table 1), and while no novel variant was non-synonymous, several were found to have bioinformatic evidence of a potential functional effect. There were two synonymous SNVs predicted to change splice site enhancers and silencers, as well as three variants in the 3′ UTR sequence.

**Table 1:** Description of novel variants identified by sequencing in *ERBB4*.

Novel variant number	Location (bp)	SNP	reference allele	new allele	minor allele frequency	location	Bioinformatic Annotation
1	213,403,857	W	T	A	0.003	5′ upstream / in unspliced EST	
2	213,403,847	S	G	C	0.003	5′ upstream / in unspliced EST	
3	212,590,021	M	A	C	0.003	intron 102 bp 5′ of exon 7	
4	212,566,810	Y	C	T	0.003	exon 12	Synonymous; Loss of exon splice enhancer
5	212,543,982	K	G	T	0.003	intron 73 bp 5′ of exon 13	
6	212,488,828	S	C	G	0.003	intron 59 bp 5′ of exon 18	
7	212,426,588	R	G	A	0.056	intron 40 bp 3′ of exon 20	Adjacent to alternative stop codon
8	212,295,591	A indel	−/−	A/-	0.247	intron 80 bp 3′ of exon 21	
9	212,293,120-2	CTT indel	CTT/CTT	CTT/-	0.003	intron 85 bp 5′ of exon 22	
10	212,286,804	R	A	G	0.003	exon 24	Synonymous; Gain of exon splice silencer
11	212,251,910	Y	C	T	0.008	intron 35 bp 5′ of exon 27	
12	212,251,550	R	G	A	0.003	intron 28 bp 3′ of exon 27	
13	212,251,537	M	C	A	0.003	intron 41 bp 3′ of exon 27	
14	212,248,283	M	C	A	0.008	3′ UTR (57 bp)	
15	212,248,281	S	C	G	0.005	3′ UTR (59 bp)	
16	212,248,219	R	G	A	0.003	3′ UTR (121 bp)	

**Figure 2:**
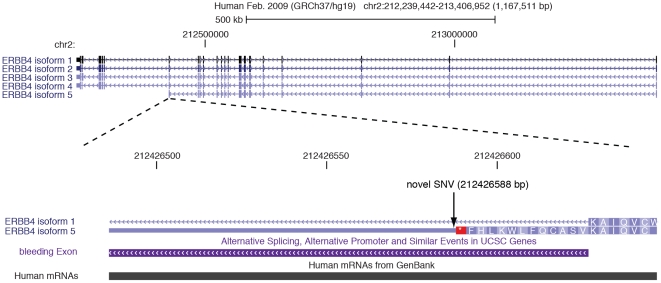
*ERBB4* gene structure with a focus on a novel variant within a “bleeding” exon 20.

Among the 16 novel variants, our power analyses indicated that only two variants (SNVs 7 and 8) had allele frequencies sufficiently high enough (MAF>0.03) to warrant additional genotyping in our available 999 independent controls. Of these, the potentially most interesting finding was SNV 7 (G>A), which was absent in dbSNP 132, but was present in 21 out of 188 cases (11.2% prevalence, MAF of 5.6%). As shown in Fig. 2, this SNV is 40 bp downstream of exon 20 and is located next to an alternate “bleeding” form of exon 20 that is associated with a prematurely truncated transcript of *ERBB4*, with no evidence of being translated (http://genome.ucsc.edu/).

To determine whether this novel SNV was associated with mood-incongruent psychotic BP, we performed an association study by genotyping additional controls (N = 999) and the remaining (non mood-incongruent psychotic) BP cases (N = 806) from the GAIN BP sample (Table 2). The novel marker was in Hardy-Weinberg equilibrium in both cases (*P*-value = 1.0) and controls (*P*-value = 0.36), and was present in 6.9% of controls, with a MAF of 3.6%. Using logistic regression with principal components as covariates we found an association of the novel SNV in cases with mood-incongruent psychotic BP compared with controls that was statistically significant in a dominant model (OR = 1.73, *P*-value = 0.039) and close to significance in an additive model (OR = 1.64, *P*-value = 0.055). This association appeared to be specific to cases with mood-incongruent psychotic BP, since a case-only analysis found an enrichment of the putative risk allele in cases with mood-incongruent psychosis compared with all other BP cases (Table 2). The second variant tested for association was a common A/- insertion-deletion polymorphism (SNV 8). This variant was genotyped in the 999 controls and 806 non-mood incongruent BP cases, but showed no evidence of association in either the case-control (*P*-value = 0.87) or the case-only analyses (*P*-value = 0.86).

**Table 2:** Association analysis of a novel SNV (chr2:212426588).

Analysis	M.A.F.	Additive Model		Dominant Model	
	(N subjects)	OR	*P*-value	OR	*P*-value
**Mood-incongruent psychotic BP**	5.6% (189)				
**vs.**	vs.	1.64	0.055	1.73	0.039
**Controls**	3.6% (999)				
**Mood-incongruent psychotic BP**	5.6% (189				
**vs.**	vs.	1.65	0.063	1.65	0.063
**All other BP cases**	3.6% (806)				

## Discussion

In this study we sequenced all coding regions of *ERBB4*, a candidate gene with strong biological plausibility, as well as suggestive evidence for genetic association with schizophrenia. We hypothesized that rare novel functional variants, potentially of large effect, would be over-represented in BP cases with mood-incongruent psychotic features, a subset of BP with phenotypic similarities to schizophrenia. Although sequencing of 188 cases revealed no evidence of a non-synonymous or loss of function variant, we identified an intriguing variant with a possible functional effect, and found an association of this variant with the mood-incongruent psychotic form of BP (dominant *P*-value = 0.039).

There are at least five alternatively spliced transcripts of *ERBB4* documented in the UCSC Genome Browser, including one transcript that ends just after exon 20 and has no associated protein product. This transcript includes a retained 140 bp sequence of intron 20 (hg 19 coordinates 212,426,487–212,426,626), which, as shown in Fig. 2, leads to the transcription of a “bleeding” exon with an alternative stop codon. The newly identified single nucleotide variant is one base downstream of the alternative stop codon and, if functional, may have an impact on transcription of the shorter and non-functional *ERBB4* isoform. Although this hypothesis remains to be experimentally validated, it raises the possibility that risk alleles may, among other mechanisms, disrupt the normal isoform “balance” of alternatively spliced genes [28], [29].

The major psychotic syndromes are likely to be heterogeneous categories with many underlying etiologies. Clinical subphenotypes like mood-incongruent psychosis may help mitigate this complexity. If the BP phenotype is composed of multiple subphenotypes with partially distinct genetic causes, then any particular genetic variant might contribute to causation of one or several subphenotypes, but not all. Subphenotypes of the disorder might be more informative for purposes like gene mapping if the increased effect size of a risk allele due to genetic homogeneity within the subgroup outweighs the reduction in power from a smaller sample size [30]. In this study, the association of a novel SNP with mood-incongruent BP yielded a larger effect size (OR∼1.6–1.7) than those typically seen in GWAS of psychiatric disorders.

This study has several limitations, perhaps the most important being the relatively small sample size. Assuming a binomial distribution, our sample of 188 sequenced cases had an 80% probability to find a mutation with a case frequency >0.4%, which is likely to miss many rare variants, particularly if extensive allelic heterogeneity is present. In our genotyped case-control sample we had 80% power to detect OR≥1.9, but only ∼55% power to detect ORs in the range found in this study (ORs≥1.6). Modest sample sizes such as these are also more likely to produce inflated effect size estimates and are vulnerable to chance findings (type I error). A further limitation which may increase type I error stems from our strategy to sequence cases, while only genotyping potentially functional variants in controls [31], although this is likely lessened by our tested variant being uncommon rather than rare, and by the control sample size being over five times larger than the case sample size. Additional limitations include the limited coverage of the promoter and the 3′ UTR sequences and the very restricted coverage of the introns. Given the length of *ERBB4* (1.16 MB) full sequencing of the gene is likely to be feasible only in the context of whole genome sequencing. Finally, we note that although the discovered SNV may potentially affect splicing, experimental validation is necessary to test this hypothesis.

In conclusion, we find no evidence of unambiguous loss of function mutations in 188 cases with mood-incongruent psychotic BP. We discovered a novel variant present in 11% of cases and 6% of controls that may have functional importance, but additional studies are necessary to replicate this association and to study the impact of the variant on splicing of *ERBB4*.

## Methods

### Ethics Statement

All samples were collected from study participants after obtaining written informed consent under clinical research protocols approved by the Johns Hopkins University School of Medicine institutional review board.

### Subjects

Cases were selected from the 1,001 BP cases and 1,033 controls of European-American descent genotyped through the GAIN consortium by the Bipolar Genome Study (BiGS) [21]. All cases were interviewed with the Diagnostic Interview for Genetic Studies (DIGS) and best-estimate diagnoses were made by two research psychiatrists or PhD psychologists. Among the BP cases, we initially selected for sequencing the 189 subjects from the GAIN BP sample who had a lifetime history of mood-incongruent psychosis as previously defined [5]. Briefly, subjects were classified as cases with mood-incongruent psychotic bipolar disorder if they had a lifetime history of running commentary auditory hallucinations, or passivity delusions such as delusions of being controlled, or delusions of thought insertion, withdrawal, or broadcasting. Subjects were also included if their psychotic symptoms during their most severe depression or mania were judged by the interviewer to be “inconsistent” with typical depressive or manic themes. Of the 189 subjects, one subject was sequenced in duplicate, and DNA for one subject was unavailable, leading to a final count of 188 subjects sequenced across the *ERBB4* gene.

In our association study we genotyped all 189 cases as well as 810 non mood-incongruent BP cases and 999 healthy controls from the GAIN BP consortium sample. These controls were previously ascertained using an Internet based adaption of the Composite International Diagnostic Interview-Short Form (CIDI-SF) [22]. Controls were selected to have no self-reported history of hallucinations, bipolar disorder, or schizophrenia, and no history of sufficient lifetime depressive symptoms to meet DSM-IV criteria for major depressive disorder.

### Sequencing

Conventional PCR amplification and Sanger sequencing were performed by Polymorphic DNA technologies Inc. (Alameda, CA, USA). Primers were designed based on the NCBI36/hg18 reference sequence of the longest *ERBB4* transcript (RefSeq NM_005235; CCDS 2394). The sequenced regions included all 28 exons, 1 kb of the promoter region, the 5′UTR, and 400 bp of the 7.9 kb 3′UTR. Sequencing was performed on both strands and chromatograms were aligned and visualized using CodonCode Aligner (CodonCode Corporation, Dedham, MA, USA). One sample was sequenced in duplicate across all PCR amplifications and showed 100% concordance. Sequencing of the promoter region was divided into five PCR amplicons; however, the first three amplicons yielded poor sequence quality in all samples and were excluded from the analysis. The remaining two amplicons (closest to the promoter) yielded approximately 600 bp of high quality sequence.

All novel single nucleotide variants (SNVs) were confirmed either with bidirectional sequencing, or, if the complimentary strand was of poor sequencing quality, with additional genotyping (see below).

### Genotyping

Genotyping was performed by pyrosequencing using the PyroMark MD system (QIAGEN). To confirm the novel variants that were seen only on one strand we genotyped nine novel SNVs, validating seven of these nine variants. In the case-control association, we genotyped 995 BP cases (189 with and 806 without mood-incongruent psychosis) and 999 controls from the BiGS study. Among this sample, 23 individuals were genotyped in duplicate and showed 100% concordance.

### Association analysis

To account for potential population stratification between cases and controls, we used Eigensoft [23] to derive principal components from the available GWAS data for all samples. Based on a scree plot, we selected the top two principal components to include as covariates in our association analysis. We performed association analyses using additive and dominant models.

### Bioinformatic Annotation

Novel variants were visualized in the UCSC genome browser (GRCh37/hg19) with all available annotation tracks. RESCUE-ESE was used to identify potential exonic splicing enhancers [24]. We queried UTR variants for disruption of miRNA binding sites with miRBase [25], and for changes in RNA secondary structure with RNAFold [26].

### Probability and power calculations

For the sequenced sample size of 188, we calculated the smallest minor allele frequency (MAF) that could be detected with a probability of 80% using an integral over a cumulative binomial distribution. Power for the association analysis of 188 cases and 999 controls was calculated using the Genetic Power Calculator [27] with the following assumptions: (1) full linkage disequilibrium with the pathogenic mutation; (2) an additive model; and (3) an α = 0.05. With the above parameters and a hypothesized effect size of OR = 2.0, our sample had 80% power to detect an association with variants of minor allele frequencies >0.03.
